# Scaling Relationships of the Structural and Rheological Behavior of Tadpole Polymer Chains in Dilute Solution Systems Using Brownian Dynamics Simulations

**DOI:** 10.3390/polym16202871

**Published:** 2024-10-11

**Authors:** Chaehyun Cho, Jun Mo Kim

**Affiliations:** Department of Chemical Engineering, Kyonggi University, 154-42 Gwanggyosan-ro, Yeongtong-gu, Suwon 16227, Kyonggi-do, Republic of Korea; chaehyun@kyonggi.ac.kr

**Keywords:** tadpole polymer, lasso polymer, Brownian dynamics, dilute solution, good solvent conditions

## Abstract

Tadpole polymers, also known as lasso polymers, feature molecular structures that combine a single ring with a single linear side branch, leading to distinct conformational, dynamical, and rheological characteristics compared to their corresponding counterparts, particularly pure linear and pure ring polymers. To elucidate the mechanisms underlying these distinctive behaviors, comprehensive mesoscopic Brownian dynamics (BD) simulations of dilute solution systems of tadpole polymers were conducted using a bead–rod chain model under both equilibrium and flow conditions. Three types of tadpole polymer chains were prepared by varying the ring-to-linear ratio within the tadpole chain and comparing them with the corresponding linear and ring chains. Depending on this ratio, tadpole polymer chains exhibit entirely different structural properties and rotational dynamics, both in equilibrium and under shear flow. As the linear proportion within the tadpole chain increased, the structural, dynamic, and rheological properties of the tadpole polymer chains became more similar to those of pure linear polymers. Conversely, with an increasing ring proportion, these properties began to resemble those of pure ring polymers. Based on these observed tendencies, a simple general scaling expression is proposed for tadpole polymer properties that integrates scaling expressions for both pure linear and pure ring polymers. Our results indicate that the conformational, dynamic, and rheological properties of tadpole polymers, as predicted by these simple scaling expressions, are in good agreement with the simulated values, a result we consider statistically significant.

## 1. Introduction

Polymer products, essential to modern life, are manufactured through various continuous flow processes, such as injection and extrusion [1,2,3,4]. In these processes, the molecular structure of the raw polymer material significantly influences the conformational, thermal, dynamic, rheological, and topological properties of the final polymer products. Consequently, it is crucial to have a comprehensive understanding of the structure-property-phenomenon relationship in polymers to effectively manufacture these products [1,2,3,4,5,6,7]. However, the vast array of polymer structures, resulting from the numerous combinations of different monomers, complicates the determination of the appropriate structure-property-phenomenon relationship for polymers.

Over the past few decades, significant advances in polymer chemistry have enabled the precise synthesis of new classes of polymers with a wide variety of molecular structures [6,7]. These new polymers exhibit conformational, thermal, dynamic, and topological properties that are distinctly different from those of conventional polymers. Ring, or cyclic, polymers—created by joining the two ends of a conventional linear polymer—are a prime example of this new class [8,9,10,11,12,13,14,15,16,17,18,19,20,21,22,23,24,25,26,27]. For instance, ring polymers display reduced intrinsic viscosity, faster relaxation times, and fewer entanglements compared to their linear counterparts [25,26,27,28,29,30,31,32,33,34,35,36,37,38,39,40,41,42,43,44,45,46,47,48,49,50,51,52]. Additionally, ring polymers exhibit dynamic behavior that cannot be accurately described by conventional linear polymer models, which are based on free-chain ends [4,6]. Consequently, theoretical, experimental, and numerical research efforts over the past few decades have focused on addressing unresolved questions related to ring polymers. Recent advances in ring synthesis [8,9,10,11,12,13,14,15,23,24,25] and purification techniques [53,54,55,56,57] have further accelerated the development of theories and models for these polymers.

Building on the basic understanding of ring polymers, more intricate molecular architectures, known as multicyclic polymers, are now being synthesized [58,59,60,61,62,63,64,65]. Multicyclic polymers combine multiple rings and linear units, allowing them to simultaneously exhibit properties associated with both pure linear and ring polymers. These polymers inherently possess the closed-loop structure of ring polymers and the free chain ends characteristic of linear polymers. Consequently, multicyclic polymers exhibit a greater degree of physicochemical diversity and unique dynamic behaviors compared to their pure linear and ring counterparts [66,67,68,69,70,71,72,73,74,75,76]. In polymer chemistry, multicyclic polymers are classified into three types—fused, spiro, and bridged—depending on the number of rings, linear side branches, and branch points [58]. Tadpole (or lasso) polymers are representative of fused-type multicyclic polymers, featuring the simplest structures, consisting of a single ring and a single linear side branch. Despite their simplicity, tadpole polymers retain the intrinsic closed-loop structure of ring polymers along with the free-chain ends of linear polymers. At present, the practical commercial application of tadpole polymers is limited and remains an object of academic curiosity because there are still many difficulties in the commercial synthesis and purification of tadpole polymers. Our expectation is that tadpole polymers, which have the dual characteristics of pure linear polymers and pure ring polymers, will facilitate the production of new commercial polymer materials with a wide range of tunable properties. As a result of decades of research efforts in biology, a wide variety of ring-shaped (or tadpole-shaped) biomacromolecules have been found in nature. At present, these biomacromolecules can be used in biomedical applications such as drug delivery, gene transfection, and bioaccumulation of tumor cells, in the fabrication of nanoparticles using biodegradable and biocompatible polymers, and in the production of dopants for commercial materials to prevent contamination [25]. Therefore, the fundamental understanding of tadpole polymers can be very useful in the interpretation of the intrinsic properties of tadpole-shaped biomacromolecules and the use of tadpole polymers in a variety of applications. For instance, lasso peptides, which function as antibiotics, resemble tadpole polymers [77,78,79,80,81]. Therefore, research on tadpole polymers is anticipated to play a crucial role in understanding the operating mechanisms of bioprotein molecules such as lasso peptides. Biomacromolecules with structures similar to tadpole polymers are abundant in nature, but not enough experimental work has been done on the intrinsic properties and behavior of tadpole-shaped polymers. Additionally, although polymer models describe the behavior of linear and ring polymers, few accurate models are available to explain the behavior of tadpole polymers.

Previous studies of tadpole-shaped polymer chains have been focused on the synthesis, purification, and characterization of tadpole-shaped polymer chains. Moreover, the nonlinear flow behavior of tadpole-shaped polymer chains can be very challenging to observe in experiments because of the instability of the flow at high flow rates due to edge fracture, wall slip, and so on. To the best of our knowledge, there is insufficient research to elucidate the nonlinear viscoelastic response of tadpole polymer chains under flow conditions. Here, we investigate the structural, dynamic, and rheological properties, and chain tumbling of the tadpole polymers from the linear viscoelastic regime to the nonlinear viscoelastic regime, which is difficult to achieve in conventional rheology experiments, and collect detailed information of tadpole polymer chains from the molecular level to the macroscopic level.

To investigate the conformational, dynamic, and rheological properties of tadpole polymers and support the development of polymer models for these polymers, comprehensive mesoscopic Brownian dynamics (BD) simulations were conducted on dilute solution systems of tadpole polymers using a bead–rod chain model under both equilibrium and flow conditions. In this study, three types of tadpole polymers were prepared by varying the ratio of ring-to-linear units within the chain to analyze the dual characteristics of tadpole polymers in comparison to pure linear and ring polymers. Notably, complex topological constraints that are currently under debate—such as intermolecular ring–ring or ring-linear threading and concatenation between rings—were deliberately excluded from this study to focus on the intrinsic static and dynamic behavior of the tadpole polymer itself.

This study revealed the impact of the ring-to-linear ratio within the tadpole chain on the conformational, dynamic, and rheological behaviors of tadpole polymer chains under flow conditions. Based on these findings, we determined a general scaling expression for each property of the tadpole polymer chain by combining the scaling expressions for the corresponding properties of pure linear and ring chains. The properties of tadpole polymers predicted by these simple scaling expressions were in excellent agreement with the simulated values.

## 2. Materials and Methods

**Figure 1:**
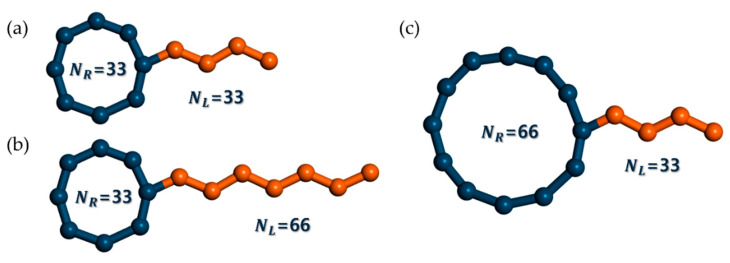
Schematic representation of three types of tadpole chain polymers designed with different ring-to-linear ratios within the chain. Note that *N_R_* and *N_L_* represent the number of beads in the ring and linear side branches, respectively. (**a**) T_R33L33 chain, with 33 beads in the ring and 33 beads in the linear side branch. (**b**) T_R33L66 chain, with 33 beads in the ring and 66 beads in the linear side branch. (**c**) T_R66L33 chain, with 66 beads in the ring and 33 beads in the linear side branch.

This study investigates the conformational, dynamic, and rheological behaviors of tadpole polymers through extensive mesoscopic Brownian dynamics (BD) simulations for dilute solution systems under both equilibrium and shear flow conditions. In these simulations, the tadpole chains in the dilute solution were modeled as isolated bead–rod chains with an implicit solvent effect. This means that the BD simulation here performs a single chain dynamics simulation. Hundreds of individual isolated test chains are simulated independently in parallel during a BD simulation. The influence of intermolecular and intramolecular interactions on the test chain is indirectly taken into account by means of hydrodynamic interaction (HI) and excluded volume (EV). Therefore, topological constraints are not conceptually possible in the BD simulation of the tadpole chain in the dilute solution system. However, we have observed the intramolecular ring-linear penetration, i.e., the linear side branch of the tadpole chain penetrating the ring part of the tadpole chain. This was a very rare event and did not persist for long periods of time. Consequently, complex topological constraints, such as intermolecular ring–ring threading, intermolecular ring-linear threading, and concatenated structures between rings, were deliberately excluded. By removing all external factors except the molecular structure, we focused on the inherent static and dynamic behaviors of tadpole polymers with the same molecular weight but different molecular architectures. As illustrated in Figure 1, we designed three types of tadpole polymers with identical molecular weights by varying the ratio of ring-to-linear units within the chain: (i) the T_R33L33 chain, with 33 beads in the ring and 33 beads in the linear side branch; (ii) the T_R66L33 chain, with 66 beads in the ring and 33 beads in the linear side branch; and (iii) the T_R33L66 chain, with 33 beads in the ring and 66 beads in the linear side branch. The ring-to-linear ratios within the chain were 1:1 for the T_R33L33 chain, 2:1 for T_R66L33, and 1:2 for T_R33L66 chains, respectively.

To obtain accurate results and facilitate comparisons with previous studies, the number of beads per chain was fixed at appropriate values (e.g., 66 or 99 beads per tadpole chain). For instance, as a single rod approximately corresponds to six CH_2_ atoms in a polyethylene (PE) molecule under shear flow, the length of a tadpole polymer chain is roughly equivalent to that of C_400_H_804_ or C_600_H_1204_ PE chains [81]. Then, we carefully selected the ratio of ring-to-linear segments within the chain by studying the molecular architectures of existing synthetic polymers and natural biomacromolecules. For example, a series of tadpole-shaped polystyrene chains with 1:1, 1:2, and 2:1 ratios of ring-to-linear units within the tadpole chain were employed to investigate the linear melt rheology of synthetic tadpole-shaped polymer chains [74]. In addition, previous studies of biomacromolecules have shown that peptides such as Microcin J25, MS-271 (siamycin I), and astexin-1, RES-701-1, and RP 71955 (siamycin II) have a lasso structure with a 1:1 ratio of ring-to-linear units within the chain [79,80,81,82]. Hundreds of identical tadpole chains, each consisting of 66 or 99 beads, were used in each BD simulation to ensure the accuracy of the results. Additionally, BD simulations were performed on dilute solution systems of pure linear and ring polymers, which were closely compared with the results for tadpole polymers. Pure linear and ring polymers are designated by the polymer type and number of beads in the chain (e.g., L33, L66, L99, R33, R66, and R99). Detailed simulation conditions for each system are shown in Table 1. Each simulation has run over several times longer than the longest relaxation time of the system.

BD simulations for dilute solution systems of tadpole polymers were conducted, incorporating hydrodynamic interaction (HI) and excluded volume (EV) effects to accurately reflect intra- and inter-molecular interactions. In the bead–rod BD simulation, the stochastic differential equation (SDE) for the evolution of the bead position, derived by Öttinger [83], is as follows:

(1) $$\begin{array}{ll} dr_{i} & =\sum_{j}P_{ij}\cdot \left\{\left[v_{0}+\kappa \cdot r_{j}+\sum_{l}D_{jl}\cdot \left(F_{l}+F_{l}^{\left(e\right)}+F_{l}^{\left(m\right)}\right)\right]dt\right.+\left.\sqrt{2k_{B}T}\sum_{l}B_{jl}\cdot dW_{l}\right\}\\ & -k_{B}T\sum_{v=1}^{d}\sum_{kjl}\left[P_{ij}\cdot D_{kl}\right]:\frac{\partial^{2}g_{v}}{\partial r_{l}\partial r_{j}}\frac{\partial R_{i}}{\partial g_{v}}dt\\ & -k_{B}T\sum_{v=1}^{d}\sum_{kjl}P_{ik}\cdot D_{kl}\cdot \frac{\partial^{2}g_{v}}{\partial r_{l}\partial r_{j}}\cdot \frac{\partial R_{i}}{\partial g_{v}}dt+k_{B}T\sum_{kjl}P_{kj}^{T}:\left(\frac{\partial }{\partial r_{k}}D_{jl}\right)\cdot P_{il}^{T}dt \end{array}$$

where **r***_i_* indicates the position vector of bead *i*, and $P_{ij}$ denotes the dynamic projection tensor. The velocity gradient κ describes the streaming velocity field of $v_{0}+\kappa \cdot r_{i}$, $D_{ij}$ and $B_{ij}$ present to the mobility tensor and the decomposed tensor of the mobility tensor, respectively, which are related to $D_{ij}=\sum_{k}B_{ik}\cdot B_{jk}^{T}$. $k_{B}$ is the Boltzmann constant and *T* is the temperature. $F_{i}$, $F_{i}^{\left(e\right)}$, and $F_{i}^{\left(m\right)}$ indicate the internal interaction, external forces, and metric forces on bead *i*, respectively. The Wiener process $W_{i}\left(t\right)$ represents a time-dependent random variable of *i-*th bead and follows a Gaussian distribution with $\langle W_{i}\left(t\right)\rangle =0$ and $\langle W_{i}\left(t\right)W_{i}\left(t^{\prime }\right)\rangle =\delta_{ij}\min(t,t^{\prime })\delta$, where δ is the unit tensor, and *g_v_* is the constraint. $R_{i}$ indicates the position vector with respect to the center of mass $R_{i}=r_{i}-r_{c}$, where $r_{c}$ is the center of mass position vector. A detailed derivation of converting the diffusion equation with generalized coordinates into an SDE can be found elsewhere [47]. In the mesoscopic bead–rod BD simulation, Equation (1) is numerically integrated using the iterative method proposed by Liu et al. [84]. The HI interaction is defined by the Rotne–Prager–Yamakawa (RPY) tensor [85,86], expressed as follows:

(2) $$D_{ij}=\left\{\begin{array}{cc} I & i=j\\ \frac{3\sigma }{4|r_{ij}|}\left[\left(1+\frac{2\sigma^{2}}{3r_{ij}^{2}}\right)I+\left(1-\frac{2\sigma^{2}}{r_{ij}^{2}}\right)\frac{r_{ij}r_{ij}^{T}}{r_{ij}^{2}}\right] & i\neq j,|r_{ij}|\;\geq \sigma \\ \left[\left(1-\frac{9|r_{ij}|}{32\sigma }\right)I+\left(\frac{3}{32\sigma |r_{ij}|}\right)r_{ij}r_{ij}^{T}\right] & i\neq j,|r_{ij}|\;<\sigma \end{array}\right.$$

where ***I*** is the second-rank unit tensor. The EV interaction is described by the Weeks-Chandler-Andersen (WCA) model [87] as follows:

(3) $$U_{LJ}(r_{ij})=\left\{\begin{array}{cc} 4\left[{\left(\frac{\sigma }{r_{ij}}\right)}^{12}-{\left(\frac{\sigma }{r_{ij}}\right)}^{6}\right] & r_{ij}\leq 2^{1/6}\sigma \\ -\;1 & r_{ij}>2^{1/6}\sigma \end{array}\right.$$

where $r_{ij}=\left|r_{ij}\right|=\left|r_{i}-r_{j}\right|$ denotes the distance between the beads and *σ* = 0.8 indicates the bead size parameter. In BD simulation, length, forces, and time are nondimensionalized using the inter-bead separation length (a), the thermal scale $\left(k_{B}T/a\right)$, and the time scale $\left(\xi a^{2}/k_{B}T\right)$, respectively, where ξ is the friction coefficient of the bead. Therefore, all the properties in this study were calculated in dimensionless (or reduced) units. The Weissenberg number (*Wi*) is defined as the product of the shear rate, $\dot{\gamma }$, and the longest rotational relaxation time, $\tau_{R}$, of the chain, i.e., $Wi=\dot{\gamma }\tau_{R}$.

## 3. Results and Discussion

### 3.1. Equilibrium

**Figure 2:**
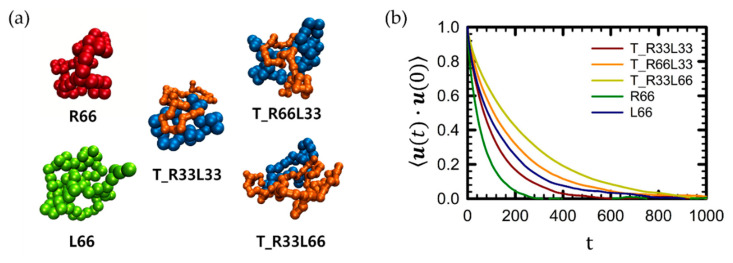
Comparison of (**a**) snapshots of a randomly selected individual chain in pure linear, pure ring, and tadpole polymer systems in equilibrium, and (**b**) time autocorrelation function (TACF) of chain unit end-to-end vector, ***u***. For tadpole polymers, the chain unit end-to-end vector ***u*** is defined as the sum of the chain ring diameter vector, **R**_d_, and the unit end-to-end vector of the linear side branch, **R**_L_.

Figure 2a shows randomly selected single chain conformations of each polymer in dilute solution at equilibrium. As illustrated in the figure, each polymer chain maintains a random coil conformation at equilibrium.

To measure the size of tadpole polymer chains in dilute solutions, we calculated the mean-square radius of gyration, $\langle R_{g}^{2}\rangle$, of the tadpole polymer chains at equilibrium and compared it with the corresponding pure linear and ring polymer chains. As expected from both theory and experimental results [3,4,5,6,7], $\langle R_{g}^{2}\rangle$ of all polymer chains increases with increasing molecular weight of the chain. Generally, this increasing trend in $\langle R_{g}^{2}\rangle$ is described by a conventional scaling law of $\langle R_{g}^{2}\rangle ∼MW^{a}$. From Table 2, the conventional scaling expressions for $\langle R_{g}^{2}\rangle$ of pure linear and ring polymer chains can be approximated as ${\langle R_{g}^{2}\rangle }_{Linear}∼0.14\times MW^{1.27}$ for pure linear chains and ${\langle R_{g}^{2}\rangle }_{Ring}∼0.07\times MW^{1.27}$ for pure ring chains. MW denotes the molecular weight or number of beads. The scaling exponents obtained from the mesoscopic BD simulation for the dilute solution systems of pure linear and pure ring chains are consistent with those from polymer theory and experiments, specifically 0.635 from BD simulations and 0.588 from polymer theory and experiments under good solvent conditions [3,4,5,6,7,88,89,90,91,92]. Additionally, the BD simulation results for pure linear and pure ring polymer chains aligned with the theoretical predictions from the ring-Rouse model [32,33,34]. According to the theoretical ring-Rouse model [32,33,34], the mean-square radius of gyration of a ring polymer chain is half that of the corresponding linear chain, that is ${\langle R_{g}^{2}\rangle }_{\mathrm{Ring}}=1/2\times {\langle R_{g}^{2}\rangle }_{\mathrm{Linear}}$. As illustrated in Table 2, the BD simulation results for pure linear and pure ring chains satisfied this relationship.

As is well established, ring polymer chains typically exhibit diminished chain dimensions relative to their corresponding linear polymer chains with identical molecular weight [32,47,48]. Therefore, tadpole polymer chains consisting of a single ring and a single linear side branch are expected to have smaller overall chain dimensions compared to corresponding pure linear chains. From Table 2, the $\langle R_{g}^{2}\rangle$ of tadpole polymer chain falls between the $\langle R_{g}^{2}\rangle$ of the corresponding pure linear and pure ring chains at the same molecular weight.

For the scaling expression of the tadpole polymer, we employed a new approach instead of deriving it directly from the simulation data. To obtain the scaling expression for the tadpole polymer, we combined the conventional scaling expressions for pure linear and pure ring chains.

(4) $${\langle R_{g}^{2}\rangle }_{Tadpole}∼k\left[{\left(\alpha_{Linear}\times MW^{a}\right)}_{Linear}+{\left(\alpha_{Ring}\times MW^{b}\right)}_{Ring}\right]$$

where the constant *k* is estimated to be 1.15 based on the $\langle R_{g}^{2}\rangle$ of the T_R33L33 chain at equilibrium. Additional variables can be extracted from the above conventional scaling expressions for pure linear chains and pure ring chains at equilibrium. We used Equation (4) to estimate the $\langle R_{g}^{2}\rangle$ for all tadpole chains and compared it with the simulated values in Table 2. Notably, the estimated ${\langle R_{g}^{2}\rangle }_{Tadpole}$ for each tadpole polymer system using Equation (4) shows good agreement with the simulated values, as depicted in Table 2.

The longest rotational relaxation time (or Rouse time), $\tau_{R}$, of the tadpole chain was estimated using the time autocorrelation function of the chain unit end-to-end vector with an exponential decay function, that is, $\langle u(t)\cdot u(0)\rangle ∼\exp\left(-t/\tau_{R}\right)$ [4,5,6]. As expected from both theoretical and experimental results [3,4,5,6,7], $\tau_{R}$ of any polymer chain increased with increasing molecular weight (e.g., $\tau_{R}∼MW^{2}$ for the melt system and $\tau_{R}∼MW^{1.5}$ for dilute solution systems). From Table 3, this increasing trend in the relaxation times of the pure linear and ring polymer chains can be expressed as $\tau_{R}^{Linear}∼0.12\times MW^{1.70}$ for pure linear chains and $\tau_{R}^{Ring}∼0.04\times MW^{1.80}$ for pure ring chains. Using these equations, we determined the scaling relationship of the relaxation time of the tadpole polymer as follows:

(5) $$\tau_{R}^{\mathrm{Tadpole}}∼k\left[{\left(\alpha_{Linear}\times MW^{a}\right)}_{Linear}+{\left(\alpha_{Ring}\times MW^{b}\right)}_{Ring}\right]$$

where the constant *k* is 1.61 from the simulation data of the T_R33L33 chain. Other variables are obtained from the above scaling expressions for pure linear chains and pure ring chains at equilibrium. As shown in Table 3, the estimated $\tau_{R}$ values for each tadpole polymer system were consistent with the $\tau_{R}$ values obtained from the BD simulations. Based on the independent contributions of the linear and ring components in the scaling equation for the tadpole polymer, it can be assumed that these components are not closely related, either structurally or dynamically.

### 3.2. Shear Flow

**Figure 3:**
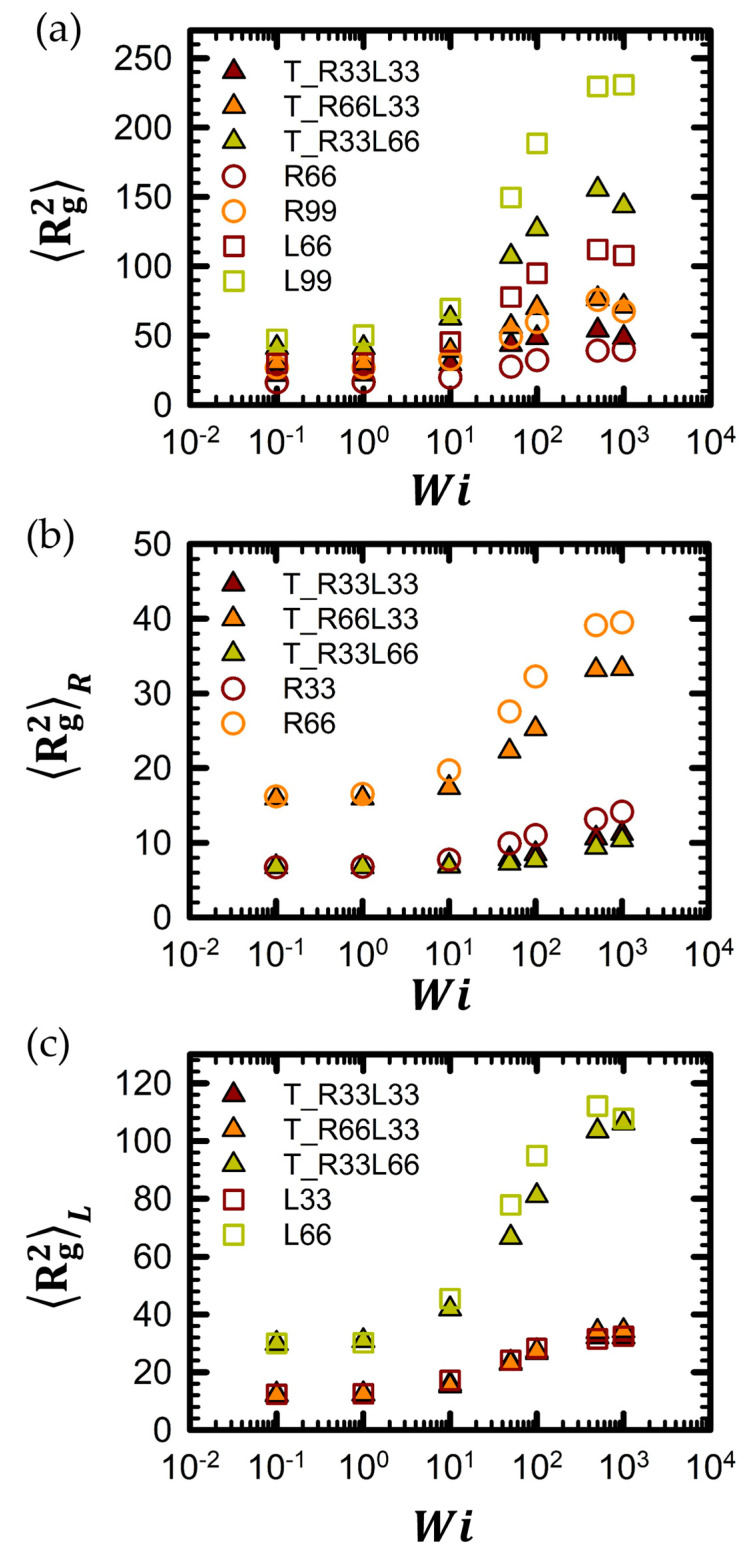
The mean-square radius of gyration, $\langle R_{g}^{2}\rangle$, of (**a**) the entire tadpole chain, (**b**) the ring part within the tadpole polymer chain, and (**c**) the linear side branch within the tadpole polymer chain as a function of *Wi*. The statistical uncertainties are represented by standard errors, which were calculated according to [95], for all simulation results. The error bars are smaller than the symbol sizes unless otherwise specified. The mean-square radius of gyration, $\langle R_{g}^{2}\rangle$, of all tadpole chain systems, as well as corresponding pure linear and ring chain systems, can be found in Figure S1 of the Supplementary Material.

To analyze the structural changes in the tadpole chains, we investigated the mean-square radius of gyration, $\langle R_{g}^{2}\rangle$, for all tadpole chains as a function of the flow field strength and compared it with the mean-square radius of gyration, $\langle R_{g}^{2}\rangle$, of the pure ring and linear chains, as shown in Figure 3. For further information regarding $\langle R_{g}^{2}\rangle$ for all tadpole chain systems and their corresponding pure linear and ring chain counterparts, refer to Figure S1 of the Supplementary Material. As depicted in the figure, the overall qualitative behavior of $\langle R_{g}^{2}\rangle$ exhibits similar trends as a function of *Wi*. For instance, the curves for all tadpole chain systems remain near equilibrium values in the low *Wi* regime, and then exhibit a sudden increase in the intermediate *Wi* regime. In the high *Wi* regime, the curves reach a plateau before decreasing as *Wi* increases further. Previous studies [82,91,93,94] have linked this characteristic response of $\langle R_{g}^{2}\rangle$ to the shear flow field to two main molecular factors: chain stretching and orientation in the low-to-intermediate *Wi* regime and chain tumbling in the high *Wi* regime.

Pure ring polymer chains generally exhibit smaller chain dimensions than their linear counterparts with the same molecular weight, both at equilibrium and under flow conditions [32,47,48]. Thus, tadpole polymer chains with a higher proportion of ring units are expected to have smaller overall chain dimensions at the same molecular weight. As shown in Figure 3a, at the same *Wi*, the overall chain dimensions of the polymer chains decrease in the following order: L99 > T_R33L66 > T_R66L33 > R99. This trend is consistent with our expectations.

For a more detailed examination of the chain dimensions, we separated the $\langle R_{g}^{2}\rangle$ of the entire chain into two components: ${\langle R_{g}^{2}\rangle }_{R}$ for the ring units and ${\langle R_{g}^{2}\rangle }_{L}$ of the linear units. These were compared with the corresponding pure linear and ring polymer chains, as shown in Figure 3b,c. Notably, the overall behavior of $\langle R_{g}^{2}\rangle$ for each component was qualitatively and quantitatively similar to that of the corresponding pure linear and ring polymer chains. This suggests that while the ring and linear parts are connected within the chain, they do not significantly influence each other structurally.

From this perspective, we derived a new scaling expression for $\langle R_{g}^{2}\rangle$ in tadpole polymers by simply adding the scaling relations of $\langle R_{g}^{2}\rangle$ for pure linear and cyclic chains.

(6) $${\langle R_{g}^{2}\rangle }_{Tadpole}∼k\left[M_{L}{\left(\alpha_{Linear}\times Wi^{a}\right)}_{Linear}+M_{R}{\left(\alpha_{Ring}\times Wi^{b}\right)}_{Ring}\right]$$

where the constant *k* is found to be 1 based on the simulation data of the T_R33L33 chain. Additional variables can be determined from simulation data pertaining to the pure L33, L66, R33, and R66 chains. Based on the conventional scaling laws for pure linear and ring chains (${\langle R_{g}^{2}\rangle }_{Linear}∼MW^{1.27}$ and ${\langle R_{g}^{2}\rangle }_{Ring}∼MW^{1.27}$), the $M_{L}$ and $M_{R}$ factors were estimated as 2.41 (=L66/L33 = $66^{1.27}/33^{1.27}$ = $2^{1.28}$) and 2.41 (=R66/L33 = $66^{1.27}/33^{1.27}$ = $2^{1.27}$), respectively. It is noted that the $\langle R_{g}^{2}\rangle$ values between *Wi* = 10 and *Wi* = 500 were used to calculate the scaling exponent of pure linear and pure ring polymer chains. In Table 4, the estimated ${\langle R_{g}^{2}\rangle }_{Tadpole}$ obtained from Equation (6) was significantly consistent with the simulated $\langle R_{g}^{2}\rangle$ obtained from BD simulations.

**Figure 4:**
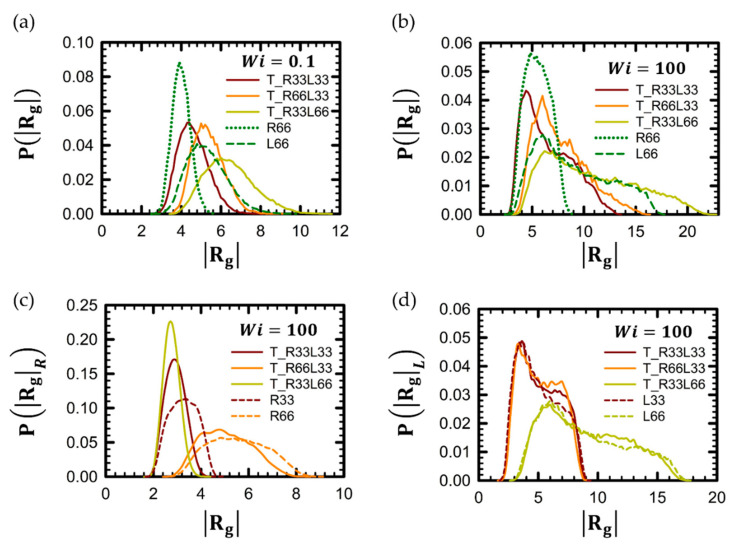
Probability distribution function (PDF) of $\left|R_{g}\right|$, $P\left(\left|R_{g}\right|\right)$, for the chain in shear flow. The $P\left(\left|R_{g}\right|\right)$ for the entire tadpole chains in both (**a**) the weak shear flow regime (*Wi* = 0.1) and (**b**) the strong shear flow regime (*Wi* = 100) is presented. Additionally, the $P\left(\left|R_{g}\right|\right)$ is provided for (**c**) the ring part and (**d**) thelinear side branch part of the tadpole polymer chain in the strong flow regime (*Wi* = 100).

The probability distribution function (PDF) of the radius of gyration, $P\left(\left|R_{g}\right|\right)$, provides detailed insights into the structural changes in polymer chains. Figure 4 presents the $P\left(\left|R_{g}\right|\right)$ for tadpole polymer chains compared with their linear and ring counterparts at both low and high *Wi*. In Figure 4a, the overall profile of $P\left(\left|R_{g}\right|\right)$ for tadpole polymer chains maintains a standard Gaussian distribution at low *Wi*, reflecting the random coil conformation of the tadpole polymer chain at near equilibrium, as shown in snapshots in Figure 2a. In contrast, $P\left(\left|R_{g}\right|\right)$ for tadpole polymer chains exhibits a distinctly non-Gaussian distribution at high *Wi*. As shown in Figure 4b, the peak of the $P\left(\left|R_{g}\right|\right)$ curve for the tadpole chain features a steep slope toward low values of $\left|R_{g}\right|$ on one side. Instead, the other side of the curve displays a steep drop followed by a gently decreasing slope toward higher values of $\left|R_{g}\right|$. These non-Gaussian behaviors are further analyzed in Figure 4c,d. From these figures, it is evident that the overall qualitative behavior of $P\left(\left|R_{g}\right|\right)$ for each part of the tadpole chain is very similar to that of $P\left(\left|R_{g}\right|\right)$ for the corresponding pure polymer chain. For example, the overall profile of $P\left(\left|R_{g}\right|\right)$ for a pure R66 chain closely resembles that of the ring unit in a T_R66L33 chain, while the overall profile of $P\left(\left|R_{g}\right|\right)$ for a pure L33 chain is similar to that of the linear unit of the T_R66L33 chain. From these results, we can infer that the overall shape of $P\left(\left|R_{g}\right|\right)$ for the tadpole chain is a straightforward combination of the distributions for pure linear and pure ring chains.

Furthermore, the shape of $P\left(\left|R_{g}\right|\right)$ for the tadpole chain appears to be influenced by its ring-to-linear ratio. For instance, the profile of $P\left(\left|R_{g}\right|\right)$ for a T_R33L66 chain, with a ring-to-linear ratio of 1:2, resembles more closely the profile of $P\left(\left|R_{g}\right|\right)$ for the linear part of the chain rather than the ring part: it features a broad peak with a lower height compared to the pure ring chain and a long, gently decreasing slope toward higher values of $\left|R_{g}\right|$. In contrast, the profile of $P\left(\left|R_{g}\right|\right)$ for the T_R66L33 chain, with a ring-to-linear ratio of 2:1, is more similar to the profile of $P\left(\left|R_{g}\right|\right)$ for the ring part of the chain: it has a peak height similar to that of the pure ring chain and a shorter, gently decreasing portion.

Notably, the peak of the ring portion in the T_R33L66 chain was higher, narrower, and shifted toward lower $\left|R_{g}\right|$ values compared to the T_R33L33 chain. This suggests that the ring part of the T_R33L66 chain is more compact than that of the T_R33L33 chain, despite both having the same molecular weight in the ring portion. Additionally, the ring part of the tadpole chain appeared more compact than the corresponding pure ring chain (e.g., T_R33L33 and T_R33L66 chains versus the pure R33 chain, as shown in Figure 4c). Conversely, the linear part of the tadpole chain seems to extend more as the molecular weight of the ring increases. This observation is evident from the fact that the height of the gently decreasing part of the $P\left(\left|R_{g}\right|\right)$ curve for the linear portion is higher compared to the pure linear analog, as seen in Figure 4d.

**Figure 5:**
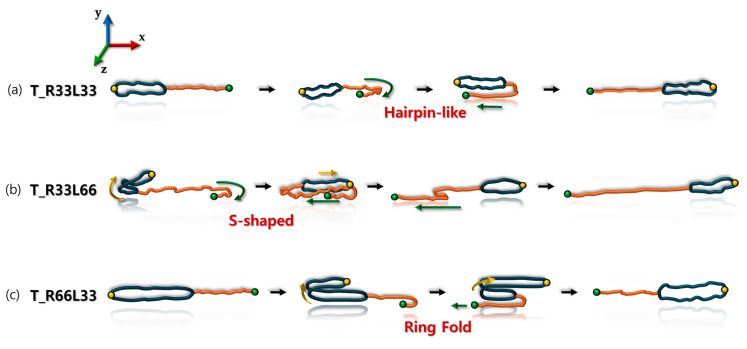
Schematic illustration of chain rotation and tumbling motions for (**a**) T_R33L33 chain, (**b**) T_R33L66 chain, and (**c**) T_R66L33 chain during a tumbling cycle under shear flow. The ring and linear side branches of the tadpole polymer chain are colored in blue and orange, respectively. The green and yellow dots serve to illustrate the complex chain rotation and tumbling behavior of the tadpole polymer chains.

Figure 5 illustrates the representative chain rotation and tumbling mechanisms for each tadpole polymer chain under shear flow. The schematic illustrates a typical chain tumbling cycle at high *Wi*. As anticipated, the chain rotation and tumbling behaviors are strongly influenced by the ring-to-linear ratio within the tadpole polymers. The T_R33L66 chain, with a ring-to-linear ratio of 1:2, exhibited S-shaped (or hairpin-like) rotational behavior similar to that of a pure linear chain during the tumbling cycle. Most chain tumbling in this system begins at the free chain end of the linear side branch. The ring portion of the T_R33L66 chain does not display the typical rotation and tumbling mechanisms of pure ring polymers. Instead, it alternates between expanded and collapsed conformations during the tumbling cycle. The T_R33L33 chain, with a ring-to-linear ratio of 1:1, also displayed rotational behavior similar to that of the T_R33L66 chain. Specifically, the T_R33L33 chain tumbled in an S-shape (or hairpin) during the chain-tumbling cycle. In contrast, the T_R66L33 chain, with a ring-to-linear ratio of 2:1, exhibited a markedly different rotational behavior compared to the T_R33L66 chain in shear flow. The ring part of the T_R66L33 chain, akin to pure ring polymer chains, tends to extend its closed-loop structure due to complex intramolecular interactions, forming a two-dimensional (2D) planar ellipsoid that lies parallel to the flow vorticity (*xz*-) plane under shear flow. In this system, most chain tumbling occurred as if a 2D planar ellipsoid of the ring part of the T_R66L33 chain was folded in half and unfolded like a piece of paper. This rotational behavior of ring folding and unfolding has been noted in previous studies [47]. The linear side branch of the T_R66L33 chain exhibited local tumbling motion, alternating between elongated and folded conformations, but seemed to follow the overall rotational motion of the ring during the chain tumbling cycle. Consequently, the rotational response of the T_R66L33 chain appears to be primarily determined by the rotational behavior of the ring part of the chain. This indicates that the rotational dynamics of the tadpole chain are strongly influenced by the ring-to-linear ratio within the tadpole polymer chain.

**Figure 6:**
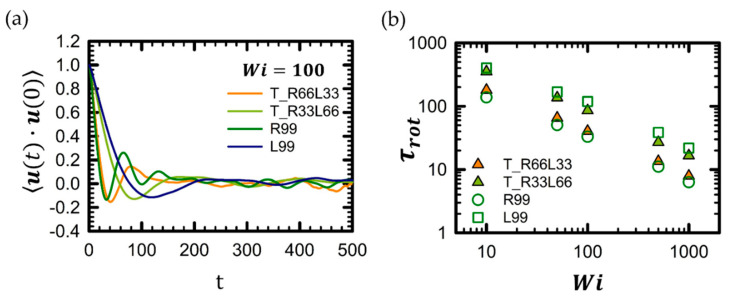
(**a**) Time autocorrelation function (TACF) of the chain unit end-to-end vector, ***u***, for the selected tadpole polymer chain system at *Wi* = 100. (**b**) Rotational time for the selected tadpole polymer chain system as a function of *Wi*. The rotational times for all tadpole chain systems and corresponding pure linear and ring chain systems can be found in Figure S2 of the Supplementary Material.

The time correlation functions (TCF) of the chain unit end-to-end vector, **u**, provide significant insights into the rotation and tumbling behavior of the chain. Figure 6a displays the time correlation function of the chain unit end-to-end vector, **u**, for the tadpole chain under strong shear flow. The chain rotational time was computed using a simple exponential decay function $\langle u(t)\cdot u(0)\rangle ∼\exp\left(-t/\tau_{rot}\right)$ for *Wi* < 10 and estimated from the characteristic negative minimum of the time correlation function for *Wi* > 10. The rotational times of the tadpole chain as a function of *Wi* are shown in Figure 6b. Generally, pure ring polymer chains exhibit faster chain rotation times compared to their linear counterparts of the same molecular weight [32,47]. Therefore, at the same molecular weight, tadpole chains with a higher proportion of ring units are expected to have faster chain rotational times.

As shown in Figure 6b, the rotational time of the tadpole chain falls between the rotational times of the corresponding pure ring and pure linear chains at each *Wi*. For instance, the rotational times of the T_R33L66 and T_R66L33 chains are situated between those of the pure R99 and pure L99 chains at each *Wi*. The rotational times increase in the order: pure R99 chain < T_R66L33 chain < T_R33L66 chain < pure L99 chain for each *Wi*. For further information regarding the rotational times for all tadpole chain systems and their corresponding pure linear and ring chain counterparts, refer to Figure S2 of the Supplementary Material.

From Table 5, the scaling expressions for the rotational time of the chain were estimated as $\tau_{rot}^{Linear}∼355.63\times Wi^{-0.71}$ for pure linear chains and $\tau_{rot}^{Ring}∼106.91\times Wi^{-0.71}$ for pure ring chains. It should be noted that for a perfectly affine chain rotation, the scaling expression of the rotational time would be $\tau_{rot}∼Wi^{-1}$. In order to establish a general scaling equation for the rotational time of the tadpole polymer, we combined the scaling expressions of rotational time for pure linear and ring chains that were mentioned above.

(7) $$\tau_{\mathrm{rot}}^{\mathrm{Tadpole}}∼k\left[M_{L}{\left(\alpha_{Linear}\times Wi^{a}\right)}_{Linear}+M_{R}{\left(\alpha_{Ring}\times Wi^{b}\right)}_{Ring}\right]$$

where the constant *k* is determined to be 1.56 based on the simulation data for the T_R33L33 chain. Additional variables can be extracted from the simulation data pertaining to the pure L33, L66, R33, and R66 chains. For example, based on the conventional scaling expressions for pure linear and ring chains (i.e., $\tau_{R}^{\mathrm{Linear}}∼MW^{1.70}$ and $\tau_{R}^{Ring}∼MW^{1.80}$), the $M_{L}$ and $M_{R}$ factors were estimated to be approximately 3.25 (=L66/L33 = $66^{1.70}/33^{1.70}$ = $2^{1.70}$) and 3.46 (=R66/L33 = $66^{1.80}/33^{1.80}$ = $2^{1.80}$), respectively. Here, the rotational time $\tau_{\mathrm{rot}}^{\mathrm{Tadpole}}$ of the tadpole chain is assumed to be the sum of the rotational times of the corresponding pure ring chain and pure linear chain. $\tau_{\mathrm{rot}}^{\mathrm{Tadpole}}$ of the tadpole chain, calculated using Equation (7), is listed in Table 5 and agrees well with the simulated rotational time obtained from the BD simulations.

**Figure 7:**
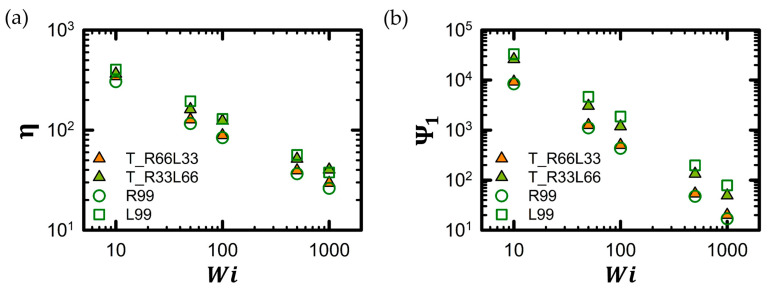
Macroscopic rheological properties of tadpole polymer chains. (**a**) Shear viscosity, η, and (**b**) first normal stress coefficient, $\Psi_{1}$, as a function of *Wi*. The shear viscosity, η, and first normal stress coefficient, $\Psi_{1}$, for all tadpole chain and corresponding pure linear and ring chain systems can be found in Figures S3 and S4 in the Supplementary Material.

Figure 7 illustrates two key macroscopic rheological properties of the tadpole chain in a dilute solution as a function of *Wi*. For further information regarding macroscopic rheological properties for all tadpole chain systems and their corresponding pure linear and ring chain counterparts, refer to Figures S3–S5 of the Supplementary Material. As shown in Figure 7a, the viscosity, η, exhibits typical shear-thinning behavior with increasing *Wi*. The power law expression $\eta ∼Wi^{-b}$ is typically used to measure the degree of shear thinning. The power law exponent, b, of the viscosity, calculated using the power law expression, shows very similar values for the pure L99 chain, the pure R99 chain, the T_R33L66 chain, and T_R66L33 chain, i.e., *b* = 0.48. This is consistent with previous findings for polymeric liquids [1,2,3,4,5,6,7,47,48,82,91,94].

Generally, the viscosity of a pure ring polymer chain is lower than that of the corresponding linear polymer chain under shear flow [32,48]. Therefore, the viscosity of the tadpole polymer chain was expected to decrease as the proportion of the ring part increased. As shown in Figure 7a, the viscosities of the polymer chains increased in the following order: pure R99 chain < T_R66L33 chain < T_R33L66 chain < pure L99 chain.

Analogous to the experimental approach of correlating structural and rheological data to scaling expressions, the scaling equations for the shear viscosity of pure linear and pure ring chains were estimated as a function of *Wi* from our simulated BD data of pure L33 chain and pure R33 chain systems. The scaling expressions for the shear viscosity were estimated as $\eta_{Linear}∼137.40\times Wi^{-0.48}$ for pure linear chains and $\eta_{Ring}∼80.17\times Wi^{-0.48}$ for pure ring chains. By simply combining these scaling expressions for the viscosities of pure linear (L33) and pure ring (R33) chains, we developed a new scaling expression for the shear viscosity of the tadpole chains as a function of *Wi*.

(8) $$\eta_{Tadpole}∼k\left[M_{L}{\left(\alpha_{Linear}\times Wi^{a}\right)}_{Linear}+M_{R}{\left(\alpha_{Ring}\times Wi^{b}\right)}_{Ring}\right]$$

where the constant *k* is found to be equal to 1.95 based on the simulation data for the T_R33L33 chain. Using the conventional scaling expressions for pure linear chains and pure ring chains (e.g., $\eta_{Linear}∼MW^{2.31}$ and $\eta_{Ring}∼MW^{2.16}$), the $M_{L}$ and $M_{R}$ factors were estimated as approximately 4.97 (=L66/L33 = $66^{2.31}/33^{2.31}$ = $2^{2.31}$) and 4.45 (=R66/R33 = $66^{2.16}/33^{2.16}$ = $2^{2.16}$), respectively. As presented in Table 6, the viscosities of the tadpole chains predicted using Equation (8) agree well with the viscosities measured through mesoscopic BD simulations as a function of *Wi*. This quantitative agreement between the predicted and measured macroscopic viscosity of the tadpole chains is notable.

The similar viscosity of the estimated and the simulated values implicitly suggests that there is a weak rheological correlation between the ring and linear segments within the tadpole polymer chains. In other words, the ring and linear segments within the tadpole polymer chain appear to behave independently from a rheological perspective. Therefore, due to the rheological independence at a higher (coarse-grained) level between ring and linear segments within the chain, the general scaling expressions consisting of pure linear and pure ring contributions can be used to predict the shear viscosity of tadpole polymer chains at any ratio of ring-to-linear segments.

Figure 7b also shows the typical shear-thinning behavior for the first normal stress coefficient, $\Psi_{1}$, for all tadpole polymer chains. The power law expression $\Psi_{1}∼Wi^{-b}$ was used to measure the degree of shear thinning for $\Psi_{1}$, and b for $\Psi_{1}$ was measured as 1.37 for pure L99 chain, 1.40 for pure R99 chain, 1.39 for T_R66L33, 1.38 for T_R33L66 chain. This agrees well with previous findings for polymeric liquids [1,2,3,4,5,6,7,47,48,82,91,94]. Similar to the macroscopic viscosity, $\Psi_{1}$ also decreases as the proportion of the ring part in the tadpole chain increases. Therefore, $\Psi_{1}$ increases in the order of pure R99 chain < T_R66L33 chain < T_R33L66 chain < pure L99 chain across the entire range of *Wi*.

To obtain a scaling expression for the $\Psi_{1}$ of the tadpole polymer chain, we employed a procedure similar to that used for macroscopic viscosity.

(9) $$\Psi_{1}^{Tadpole}∼k\left[M_{L}{\left(\alpha_{Linear}\times Wi^{a}\right)}_{Linear}+M_{R}{\left(\alpha_{Ring}\times Wi^{b}\right)}_{Ring}\right]$$

The variables in the equation were evaluated from the pure L33, L66, R33, and R66 chain results. From our simulated data of pure L33 chain and pure R33 chain systems, we estimated the scaling expressions of the $\Psi_{1}$ for pure linear and pure ring chains as $\Psi_{1}^{Linear}∼9772.37\times Wi^{-1.32}$ for pure linear chains and $\Psi_{1}^{Ring}∼1862.01\times Wi^{-1.28}$ for pure ring chains. The $M_{L}$ and $M_{R}$ factors can be derived from the conventional scaling expressions for pure linear and ring chains, i.e., $\Psi_{1}^{Linear}∼MW^{3.92}$ and $\Psi_{1}^{Ring}∼MW^{4.15}$. They can be estimated as 15.13 (=L66/L33 = $66^{3.92}/33^{3.92}$ = $2^{3.92}$) and 17.72 (=R66/L33 = $66^{4.15}/33^{4.15}$ = $2^{4.15}$), respectively. As listed in Table 7, the $\Psi_{1}$ of the tadpole chains, predicted using Equation (9), is consistent with $\Psi_{1}$ for the tadpole chain measured using the coarse-grained BD simulations as a function of *Wi*.

Under both equilibrium and flow conditions, the structural, dynamic (or chain rotational), and rheological behavior of the tadpole polymer chain can be expressed as a simple sum of the pure linear contribution and the pure ring contribution. This implicitly means that the ring and linear segments within the tadpole polymer chains are not tightly coupled, either structurally or dynamically. The physical significance behind the observed trends can be speculated as follows: In the dilute solution, where direct intermolecular interactions and topological constraints are absent, the ring and linear segments within the tadpole polymer chain appear to behave independently at some higher (coarse-grained) level, unaware of each other’s presence, despite the physical connection within the chain. Based on the above conjecture, it can be hypothesized that the unentangled tadpole polymer chains in the dilute solution system would exhibit behavior similar to that of the unentangled linear/ring blend system. Therefore, it would be very interesting to directly compare dilute solutions of tadpole polymer chains with melt solutions of unentangled linear/ring blend systems. Instead, we compared our dilute solutions of the tadpole polymer chains with the melt solutions of the entangled linear/ring blend system obtained from the experiments in Figure S6.

## 4. Conclusions

This study conducted comprehensive mesoscopic BD simulations of tadpole polymer chains in dilute solutions (or good solvent conditions) under equilibrium and shear flow conditions using the bead–rod chain model. The results were compared with those of the corresponding pure linear polymer chain and pure ring polymer chain. We modeled test chains in dilute solutions as isolated bead–rod chains with an implicit solvent effect, intentionally excluding controversial topological constraints such as intermolecular ring–ring or ring-linear threading. A tadpole polymer chain with a bimolecular structure consisting of a single ring and a single linear side branch was chosen as the test chain because it has the simplest structure among multicyclic polymers. To analyze the structure-property-phenomenon relationship, particularly the dual properties of tadpole polymers with respect to pure linear and ring polymer chains, we tested three types of tadpole polymers with the same molecular weight but different ring-to-linear ratios in the chain.

At equilibrium, the conformational and dynamic behaviors of the tadpole polymer chains in dilute solutions were consistent with previous theoretical, experimental, and numerical results. For instance, tadpole polymer chains exhibit typical scaling behavior for each property as a function of molecular weight, with the scaling exponents displaying values in good agreement with theoretical and experimental findings. The tadpole polymer chains show varying conformational and dynamic properties depending on the ring-to-linear ratio. Specifically, as the ring proportion within the chain increases, the chain size of the tadpole chain decreases, and the relaxation time becomes faster. Interestingly, the scaling expression for each property of the tadpole polymer chain can be represented as a simple sum of the scaling expressions for the corresponding pure linear chain and pure ring chain.

The conformational, dynamic, and rheological behaviors of the tadpole polymer chains in dilute solutions under flow conditions are strongly influenced by the ring-to-linear ratio within the tadpole polymer chain. Similar to the equilibrium case, as the ring proportion within the tadpole chain increases, the chain size decreases at each *Wi*, and the rotational time becomes faster compared to that of its pure linear counterparts. The tadpole polymer chains exhibit different chain rotations and tumbling behaviors depending on the ring-to-linear ratio. When the linear proportion increases, the tadpole chains display an S-shaped (or hairpin-like) rotation similar to that of the pure linear chain during the chain tumbling cycle. Conversely, as the ring proportion increases, the tadpole chains show ring folding and unfolding tumbling behaviors, akin to those of the pure ring chain. The macroscopic rheological properties, such as viscosity and the first normal stress coefficient, also vary with the ring-to-linear ratio. Both viscosity and the first normal stress coefficient decrease with increasing ring proportion within the tadpole polymer chain at each *Wi*.

We aimed to construct a general functional form using scaling laws to quantify the effect of the ring-to-linear ratio on each property. Under flow conditions, a general scaling expression for each property of the tadpole polymer chain is expressed as a function of flow strength rather than molecular weight. To develop this general scaling expression, we combined the scaling expressions for the pure linear and pure ring chains and included a term that describes the ring-to-linear ratio within the tadpole chain. The predicted values for the tadpole polymer chain properties, obtained using these general scaling expressions, were in excellent agreement—both qualitatively and quantitatively—with the data from BD simulations. We consider this result to be statistically significant.

The present findings provide an important framework for studying other types of multicyclic polymer chains, such as theta-shaped polymer chains, manacle-shaped chains, and 8-shaped polymer chains, and for designing new ring-shaped (or multicyclic) polymers and materials for specific purposes. In addition, the findings are expected to be very useful in exploiting the duality of tadpole chain polymers to adapt existing polymers and materials for specific purposes. For example, tadpole polymer can be added as an additive to the synthesis process of a conventional polymer or materials to adjust the viscosity of the conventional polymer for its intended use. By controlling the diffusion rate of the tadpole polymer chain or lasso peptide used as the drug by adjusting the ratio of ring-to-linear segments within the chain, the drug delivery time can be efficiently controlled to ensure that the drug is delivered to the desired site. One of the important potentials of this study is that it can contribute to a better understanding of the impact of complex topological constraints for the ring-shaped systems, such as intermolecular ring–ring or intermolecular linear-ring threading, on the physical and dynamical properties of ring-shaped polymers. For instance, comparing the results of an unentangled tadpole polymer chain with no topological constraints to the results of an entangled tadpole polymer chain or an entangled linear/ring blend system with topological constraints implicitly reveals the role of intermolecular ring-linear threading in tadpole polymer systems or linear/ring blend systems. Therefore, it would be very interesting to directly compare unentangled tadpole polymer chains with entangled tadpole polymer chains or entangled linear/ring blend systems.

## Figures and Tables

**Table 1:** Simulation conditions of mesoscopic bead–rod systems for the pure linear chain, the pure ring chain, and the tadpole polymer chain systems.

Chain Molecules	Pure L99	Pure R99	T_R33L33	T_R66L33	T_R33L66
No. of Beads	99	99	66	99	99
No. of Rods	98	99	65	98	98
No. of Molecules	100	100	100	100	100
Timestep	0.0005	0.0005	0.0005	0.0005	0.0005
Relaxation Time	321.75	152.03	110.84	168.52	286.13
Simulation Time	1500	1000	500	1000	1000

**Table 2:** The mean-square radius of gyration, $\langle R_{g}^{2}\rangle$, of the pure ring chain, pure linear chain, and tadpole chain at equilibrium.

Chain Length	Pure Ring		Pure Linear		Tadpole	
	$\langle R_{g}^{2}\rangle$ Simulated	$\langle R_{g}^{2}\rangle$ Estimated	$\langle R_{g}^{2}\rangle$ Simulated	$\langle R_{g}^{2}\rangle$ Estimated	$\langle R_{g}^{2}\rangle$ Simulated	$\langle R_{g}^{2}\rangle$ Estimated
33	6.73	6.58	12.23	12.25		
66	16.27	15.65	29.83	29.69	21.74 (T_R33L33)	21.64 (T_R33L33)
99	26.75	25.97	49.70	49.82	29.40 (T_R66L33)	32.07 (T_R66L33)
					42.94 (T_R33L66)	41.68 (T_R33L66)

**Table 3:** The longest rotational relaxation time (or Rouse time), $\tau_{R}$, of the pure linear chain, pure ring chain, and tadpole polymer chain at equilibrium.

Chain Length	Pure Ring		Pure Linear		Tadpole	
	$\tau_{R}$ Simulated	$\tau_{R}$ Estimated	$\tau_{R}$ Simulated	$\tau_{R}$ Estimated	$\tau_{R}$ Simulated	$\tau_{R}$ Estimated
33	21.12	20.92	48.81	47.59		
66	71.02	72.41	144.14	154.61	110.84 (T_R33L33)	110.51 (T_R33L33)
99	152.02	149.69	321.75	308.02	168.52 (T_R66L33)	193.55 (T_R66L33)
					286.13 (T_R33L66)	283.13 (T_R33L66)

**Table 4:** The mean-square radius of gyration, $\langle R_{g}^{2}\rangle$, of tadpole chains in shear flow.

*Wi*	T_R33L33		T_R66L33		T_R33L66	
	$\langle R_{g}^{2}\rangle$ Simulated	$\langle R_{g}^{2}\rangle$ Estimated	$\langle R_{g}^{2}\rangle$ Simulated	$\langle R_{g}^{2}\rangle$ Estimated	$\langle R_{g}^{2}\rangle$ Simulated	$\langle R_{g}^{2}\rangle$ Estimated
10	29.78	29.89	39.28	42.97	66.24	58.97
50	43.55	40.99	56.40	57.74	109.65	82.03
100	48.43	46.98	70.12	65.62	131.84	94.60
500	53.87	64.60	76.46	88.87	157.68	131.82

**Table 5:** Rotational time of tadpole polymer chains in shear flow.

Wi	T_R33L33		T_R66L33		T_R33L66	
	$\tau_{\mathrm{rot}}$ Simulated	$\tau_{\mathrm{rot}}$ Estimated	$\tau_{\mathrm{rot}}$ Simulated	$\tau_{\mathrm{rot}}$ Estimated	$\tau_{\mathrm{rot}}$ Simulated	$\tau_{\mathrm{rot}}$ Estimated
10	130	139.60	180	220.00	352.5	380.10
50	44.5	44.29	65.5	70.01	136.5	120.38
100	28.5	27.01	40.5	42.76	85.5	73.37
500	9	8.57	13.5	13.61	27	23.24
1000	4.9	5.23	7.9	8.31	16.6	14.16

**Table 6:** Shear viscosity of tadpole polymer chains in shear flow.

Wi	T_R33L33		T_R66L33		T_R33L66	
	η Simulated	η Estimated	η Simulated	η Estimated	η Simulated	η Estimated
10	135.19	139.03	344.26	318.89	342.66	483.67
50	64.63	63.75	127.89	147.14	170.52	220.52
100	45.74	45.57	88.66	105.46	116.62	157.24
500	21.35	20.90	39.47	48.66	55.45	71.69
1000	14.72	14.94	29.58	34.88	39.19	51.12

**Table 7:** First normal stress coefficient, $\Psi_{1}$, of tadpole polymer chains in shear flow.

Wi	T_R33L33		T_R66L33		T_R33L66	
	$\Psi_{1}$ Simulated	$\Psi_{1}$ Estimated	$\Psi_{1}$ Simulated	$\Psi_{1}$ Estimated	$\Psi_{1}$ Simulated	$\Psi_{1}$ Estimated
10	3087.92	2997.25	9225.14	11,605.31	23,713.02	38,072.64
50	386.85	361.01	1254.75	1452.79	3226.67	4539.31
100	154.75	145.12	517.43	593.77	1227.97	1816.41
500	17.60	17.49	53.69	74.40	135.67	216.58
1000	6.68	7.03	20.11	30.42	53.09	86.67

## Data Availability

Data is contained within the article or Supplementary Materials.

## Supplementary Materials

The following supporting information can be downloaded at: https://www.mdpi.com/article/10.3390/polym16202871/s1, Figure S1: The mean square radius of gyration for all chain configurations; Figure S2: The rotation time as a function of *Wi*; Figure S3: The shear viscosity of tadpole, linear, and ring chain; Figure S4: The first normal stress coefficient vs *Wi*; Figure S5: Comparison of the reduced viscosity obtained from simulation and experiment; Figure S6: (a) Steady-state viscosity of PS rings, linear and blends vs. shear rates; (b) Steady-state viscosity normalized with the zero-shear viscosity as a function of *Wi*. Refs. [48,74,96,97,98] are included in Supplementary Materials.

## References

[1] Macosko C.W. (1994). Rheology: Principles, Measurements, and Applications.

[2] Morrison F.A. (2001). Understanding Rheology.

[3] Bird R.B., Armstrong R.C., Hassager O. (1987). Dynamics of Polymeric Liquids.

[4] Doi M., Edward S.F. (1986). The Theory of Polymer Dynamics.

[5] De Gennes P.G. (1979). Scaling Concepts in Polymer Physics.

[6] Rubinstein M., Colby R.H. (2003). Polymer Physics.

[7] Flory P.J. (1953). Principles of Polymer Chemistry.

[8] Hild G., Kohler A., Rempp P. (1980). Synthesis of Ring-shaped Macromolecules. Eur. Polym. J..

[9] Hild G., Strazielle C., Rempp P. (1983). Cyclic Macromolecules—Synthesis and Characterization of Ring-Shaped Polystyrenes. Eur. Polym. J..

[10] Semlyen J.A. (2002). Cyclic Polymers.

[11] Roovers J., Toporowski P.M. (1983). Synthesis of High Molecular-Weight Ring Polystyrenes. Macromolecules.

[12] Roovers J., Toporowski P.M. (1988). Synthesis and characterization of ring polybutadienes. J. Polym. Sci. B.

[13] Kruteva M., Allgaier J., Richter D. (2023). Topology Matters: Conformation and Microscopic Dynamics of Ring Polymers. Macromolecules.

[14] Micheletti C., Chubak I., Orlandini E., Smrek J. (2024). Topology-Based Detection and Tracking of Deadlocks Reveal Aging of Active Ring Melts. ACS Macro Lett..

[15] Ha T.Y., Jeong S.H., Baig C. (2024). Interfacial Polymer Rheology of Entangled Short-Chain Branched Ring Melts in Shear Flow. Macromolecules.

[16] Peponaki K., Tsalikis D.G., Patelis N., Sakellariou G., Chang T., Vlassopoulos D. (2024). Revisiting the Viscosity of Moderately Entangled Ring Polymer Melts. Macromolecules.

[17] Ubertini M.A., Rosa A. (2023). Topological Analysis and Recovery of Entanglements in Polymer Melts. Macromolecules.

[18] Dehaghani Z.A., Chiarantoni P., Micheletti C. (2023). Topological Entanglement of Linear Catenanes: Knots and Threadings. ACS Macro Lett..

[19] Kruteva M., Monkenbusch M., Allgaier J., Pyckhout-Hintzen W., Porcar L., Richter D. (2023). Structure of Polymer Rings in Linear Matrices: SANS Investigation. Macromolecules.

[20] Doi Y. (2024). A Review of Rheology for Ring Polymers and Polymers with Internal Loops: Comparison of Recent Experimental and Theoretical Studies. Korea-Aust. Rheol. J..

[21] Vigil D.L., Ge T., Rubinstein M., O’Connor T.C., Grest G.S. (2024). Measuring Topological Constraint Relaxation in Ring-Linear Polymer Blends. Phys. Rev. Lett..

[22] Staňo R., Likos C.N., Smrek J. (2022). To Thread or Not to Thread? Effective Potentials and Threading Interactions between Asymmetric Ring Polymers. Soft Matter.

[23] Laurent B.A., Grayson S.M. (2009). Synthetic Approaches for the Preparation of Cyclic Polymers. Chem. Soc. Rev..

[24] Wang T.W., Golder M.R. (2021). Advancing Macromolecular Hoop Construction: Recent Developments in Synthetic Cyclic Polymer Chemistry. Polym. Chem..

[25] Haque F.M., Grayson S.M. (2020). The Synthesis, Properties and Potential Applications of Cyclic Polymers. Nat. Chem..

[26] Yamamoto T. (2013). Synthesis of Cyclic Polymers and Topology Effects on Their Diffusion and Thermal Properties. Polym. J..

[27] Ogawa T., Nakazono K., Aoki D., Uchida S., Takata T. (2015). Effective Approach to Cyclic Polymer from Linear Polymer: Synthesis and Transformation of Macromolecular [1]Rotaxane. ACS Macro Lett..

[28] Halverson J.D., Lee W.B., Grest G.S., Grosberg A.Y., Kremer K. (2011). Molecular Dynamics Simulation Study of Nonconcatenated Ring Polymers in a Melt. I. Statics. J. Chem. Phys..

[29] Tsamopoulos A.J., Katsarou A.F., Tsalikis D.G., Mavrantzas V.G. (2019). Shear Rheology of Unentangled and Marginally Entangled Ring Polymer Melts from Large-Scale Nonequilibrium Molecular Dynamics Simulations. Polymers.

[30] Hsiao K.W., Schroeder C.M., Sing C.E. (2016). Ring Polymer Dynamics Are Governed by a Coupling between Architecture and Hydrodynamic Interactions. Macromolecules.

[31] Young C.D., Qian J.R., Marvin M., Sing C.E. (2019). Ring Polymer Dynamics and Tumbling-Stretch Transitions in Planar Mixed Flows. Phys. Rev. E.

[32] Tsolou G., Stratikis N., Baig C., Stephanou P.S., Mavrantzas V.G. (2010). Melt Structure and Dynamics of Unentangled Polyethylene Rings: Rouse Theory, Atomistic Molecular Dynamics Simulation, and Comparison with the Linear Analogues. Macromolecules.

[33] Wiest J.M., Burdette S.R., Liu T.W., Bird R.B. (1987). Effect of Ring Closure on Rheological Behavior. J. Non-Newton. Fluid Mech..

[34] Watanabe H., Inoue T., Matsumiya Y. (2006). Transient Conformational Change of Bead-Spring Ring Chain during Creep Process. Macromolecules.

[35] Vettorel T., Grosberg A.Y., Kremer K. (2009). Statistics of Polymer Rings in the Melt: A Numerical Simulation Study. Phys. Biol..

[36] Arrighi V., Gagliardi S., Dagger A.C., Semlyen J.A., Higgins J.S., Shenton M.J. (2004). Conformation of Cyclics and Linear Chain Polymers in Bulk by SANS. Macromolecules.

[37] Sakaue T. (2012). Statistics and Geometrical Picture of Ring Polymer Melts and Solutions. Phys. Rev. E.

[38] Brown S., Lenczycki T., Szamel G. (2001). Influence of Topological Constraints on the Statics and Dynamics of Ring Polymers. Phys. Rev. E.

[39] Pasquino R., Vasilakopoulos T.C., Jeong Y.C., Lee H., Rogers S., Sakellariou G., Allgaier J., Takano A., Brás A.R., Chang T. (2013). Viscosity of Ring Polymer Melts. ACS Macro Lett..

[40] Huang Q., Ahn J., Parisi D., Chang T., Hassager O., Panyukov S., Rubinstein M., Vlassopoulos D. (2019). Unexpected Stretching of Entangled Ring Macromolecules. Phys. Rev. Lett..

[41] Liebetreu M., Likos C.N. (2020). Hydrodynamic Inflation of Ring Polymers under Shear. Commun. Mater..

[42] Milner S.T., Newhall J.D. (2010). Stress Relaxation in Entangled Melts of Unlinked Ring Polymers. Phys. Rev. Lett..

[43] Kapnistos M., Lang M., Vlassopoulos D., Pyckhout-Hintzen W., Richter D., Cho D., Chang T., Rubinstein M. (2008). Unexpected Power-Law Stress Relaxation of Entangled Ring Polymers. Nat. Mater..

[44] Rosa A., Everaers R. (2014). Ring Polymers in the Melt State: The Physics of Crumpling. Phys. Rev. Lett..

[45] O’Connor T.C., Ge T., Rubinstein M., Grest G.S. (2020). Topological Linking Drives Anomalous Thickening of Ring Polymers in Weak Extensional Flows. Phys. Rev. Lett..

[46] Chen W., Chen J., An L. (2013). Tumbling and Tank-Treading Dynamics of Individual Ring Polymers in Shear Flow. Soft Matter.

[47] Jeong S.H., Cho S., Roh E.J., Ha T.Y., Kim J.M., Baig C. (2020). Intrinsic Surface Characteristics and Dynamic Mechanisms of Ring Polymers in Solution and Melt under Shear Flow. Macromolecules.

[48] Yoon J., Kim J., Baig C. (2016). Nonequilibrium Molecular Dynamics Study of Ring Polymer Melts under Shear and Elongation Flows: A Comparison with Their Linear Analogs. J. Rheol..

[49] Roovers J. (1984). Melt Properties of Ring Polystyrenes. Macromolecule.

[50] Tsalikis D.G., Mavrantzas V.G., Vlassopoulos D. (2016). Analysis of Slow Modes in Ring Polymers: Threading of Rings Controls Long-Time Relaxation. ACS Macro Lett..

[51] Obukhov S.P., Rubinstein M., Duke T. (1994). Dynamics of a Ring Polymer in a Gel. Phys. Rev. Lett..

[52] Smrek J., Kremer K., Rosa A. (2019). Threading of Unconcatenated Ring Polymers at High Concentrations: Double-Folded *vs.* Time-Equilibrated Structures. ACS Macro Lett..

[53] Takano A., Kushida Y., Aoki K., Masuoka K., Hayashida K., Cho D., Kawaguchi D., Matsushita Y. (2007). HPLC Characterization of Cyclization Reaction Product Obtained by End-to-End Ring Closure Reaction of a Telechelic Polystyrene. Macromolecules.

[54] Singla S., Zhao T., Beckham H.W. (2003). Purification of Cyclic Polymers Prepared from Linear Precursors by Inclusion Complexation of Linear Byproducts with Cyclodextrins. Macromolecules.

[55] Noda T., Doi Y., Ohta Y., Takata S.-I., Takano A., Matsushita Y. (2020). Preparation, Characterization, and Dilute Solution Properties of Four-Branched Cage-Shaped Poly(Ethylene Oxide). J. Polym. Sci..

[56] Hövelmann C.H., Gooßen S., Allgaier J. (2017). Scale-Up Procedure for the Efficient Synthesis of Highly Pure Cyclic Poly(Ethylene Glycol). Macromolecules.

[57] Sawayama T., Wang Y., Watanabe T., Takayanagi M., Yamamoto T., Hosono N., Uemura T. (2021). Metal-Organic Frameworks for Practical Separation of Cyclic and Linear Polymers. Angew. Chem. Int. Ed..

[58] Hadjichristidis N., Hirao A., Tezuka Y., Du Prez F. (2011). Complex Macromolecular Architectures: Synthesis, Characterization, and Self-Assembly.

[59] Doi Y., Ohta Y., Nakamura M., Takano A., Takahashi Y., Matsushita Y. (2013). Precise Synthesis and Characterization of Tadpole-Shaped Polystyrenes with High Purity. Macromolecules.

[60] Doi Y., Iwasa Y., Watanabe K., Nakamura M., Takano A., Takahashi Y., Matsushita Y. (2016). Synthesis and Characterization of Comb-Shaped Ring Polystyrenes. Macromolecules.

[61] Tezuka Y., Tsuchitani A., Yoshioka Y., Oike H. (2003). Synthesis of θ-Shaped Poly(THF) by Electrostatic Self-Assembly and Covalent Fixation with Three-Armed Star Telechelics Having Cyclic Ammonium Salt Groups. Macromolecules.

[62] Polymeropoulos G., Zapsas G., Ntetsikas K., Bilalis P., Gnanou Y., Hadjichristidis N. (2017). 50th Anniversary Perspective: Polymers with Complex Architectures. Macromolecules.

[63] Kusuyama N., Daito Y., Kubota H., Kametani Y., Ouchi M. (2021). Construction of Ring-Based Architectures: Via Ring-Expansion Cationic Polymerization and Post-Polymerization Modification: Design of Cyclic Initiators from Divinyl Ether and Dicarboxylic Acid. Polym. Chem..

[64] Alkayal N., Zhang Z., Bilalis P., Gnanou Y., Hadjichristidis N. (2017). Polyethylene-Based Tadpole Copolymers. Macromol. Chem. Phys..

[65] Segawa Y., Kuwayama M., Itami K. (2020). Synthesis and Structure of [9]Cycloparaphenylene Catenane: An All-Benzene Catenane Consisting of Small Rings. Org. Lett..

[66] Uehara E., Deguchi T. (2018). Statistical Properties of Multi-Theta Polymer Chains. J. Phys. A Math. Theor..

[67] Abreu C.R.A., Escobedo F.A. (2005). A Novel Configurational-Bias Monte Carlo Method for Lattice Polymers: Application to Molecules with Multicyclic Architectures. Macromolecules.

[68] Zhu L., Wang X., Li J., Wang Y. (2016). Radius of Gyration, Mean Span, and Geometric Shrinking Factors of Bridged Polycyclic Ring Polymers. Macromol. Theory. Simul..

[69] Pipertzis A., Hossain M.D., Monteiro M.J., Floudas G. (2018). Segmental Dynamics in Multicyclic Polystyrenes. Macromolecules.

[70] Doi Y., Takano A., Takahashi Y., Matsushita Y. (2021). Viscoelastic Properties of Dumbbell-Shaped Polystyrenes in Bulk and Solution. Macromolecules.

[71] Haydukivska K., Blavatska V., Paturej J. (2023). Molecular Conformations of Dumbbell-Shaped Polymers in Good Solvent. Phys. Rev. E.

[72] Doi Y., Takano A., Takahashi Y., Matsushita Y. (2022). Terminal Relaxation Behavior of Entangled Linear Polymers Blended with Ring and Dumbbell-Shaped Polymers in Melts. Rheol. Acta.

[73] Murashima T., Hagita K., Kawakatsu T. (2022). Topological Transition in Multicyclic Chains with Structural Symmetry Inducing Stress-Overshoot Phenomena in Multicyclic/Linear Blends under Biaxial Elongational Flow. Macromolecules.

[74] Doi Y., Takano A., Takahashi Y., Matsushita Y. (2015). Melt Rheology of Tadpole-Shaped Polystyrenes. Macromolecules.

[75] Rosa A., Smrek J., Turner M.S., Michieletto D. (2020). Threading-Induced Dynamical Transition in Tadpole-Shaped Polymers. ACS Macro Lett..

[76] Doi Y., Takano A., Takahashi Y., Matsushita Y. (2020). Melt Rheology of Tadpole-Shaped Polystyrenes with Different Ring Sizes. Soft Matter.

[77] Golba B., Benetti E.M., De Geest B.G. (2021). Biomaterials Applications of Cyclic Polymers. Biomaterials.

[78] Niemyska W., Dabrowski-Tumanski P., Kadlof M., Haglund E., Sułkowski P., Sulkowska J.I. (2016). Complex Lasso: New Entangled Motifs in Proteins. Sci. Rep..

[79] Wilson K.A., Kalkum M., Ottesen J., Yuzenkova J., Chait B.T., Landick R., Muir T., Severinov K., Darst S.A. (2003). Structure of Microcin J25, a Peptide Inhibitor of Bacterial RNA Polymerase, Is a Lassoed Tail. J. Am. Chem. Soc..

[80] Zong C., Maksimov M.O., Link A.J. (2016). Construction of Lasso Peptide Fusion Proteins. ACS Chem. Biol..

[81] Katahira R., Yamasaki M., Matsuda Y., Yoshida M. (2016). MS-271, A Novel Inhibitor of Calmodulin-activated Myosin Light Chain Kinase from Streptomyces sp.—II. Solution Structure of MS-271: Characteristic Features of the ‘Lasso’ Structure. Bioorg. Med. Chem..

[82] Kim J.M., Edwards B.J., Keffer D.J., Khomami B. (2010). Dynamics of Individual Molecules of Linear Polyethylene Liquids under Shear: Atomistic Simulation and Comparison with a Free-Draining Bead-Rod Chain. J. Rheol..

[83] Öttinger H.C. (1996). Stochastic Processes in Polymeric Fluids.

[84] Liu T.W. (1989). Flexible Polymer Chain Dynamics and Rheological Properties in Steady Flows. J. Chem. Phys..

[85] Rotne J., Prager S. (1969). Variational Treatment of Hydrodynamic Interaction in Polymers. J. Chem. Phys..

[86] Yamakawa H. (1970). Transport Properties of Polymer Chains in Dilute Solution: Hydrodynamic Interaction. J. Chem. Phys..

[87] Weeks J.D., Chandler D., Andersen H.C. (1971). Role of Repulsive Forces in Determining the Equilibrium Structure of Simple Liquids. J. Chem. Phys..

[88] Schroeder C.M., Teixeira R.E., Shaqfeh E.S.G., Chu S. (2005). Characteristic Periodic Motion of Polymers in Shear Flow. Phys. Rev. Lett..

[89] Teixeira R.E., Babcock H.P., Shaqfeh E.S.G., Chu S. (2005). Shear Thinning and Tumbling Dynamics of Single Polymers in the Flow-Gradient Plane. Macromolecules.

[90] Teixeira R.E., Dambal A.K., Richter D.H., Shaqfeh E.S.G., Chu S. (2007). The Individualistic Dynamics of Entangled DNA in Solution. Macromolecules.

[91] Cho S., Kim J.M., Baig C. (2020). Scaling Characteristics of Rotational Dynamics and Rheology of Linear Polymer Melts in Shear Flow. Macromolecules.

[92] Jeong S.H., Cho S., Baig C. (2023). Chain Rotational Dynamics in Dilute Polymer Solutions and Melts under Shear Flow. Polymer.

[93] Moore J.D., Cui S.T., Cochran H.D., Cummings P.T. (2000). A Molecular Dynamics Study of a Short-Chain Polyethylene Melt. I. Steady-State Shear. J. Non-Newton. Fluid Mech..

[94] Kim J.M., Keffer D.J., Kröger M., Edwards B.J. (2008). Rheological and Entanglement Characteristics of Linear-Chain Polyethylene Liquids in Planar Couette and Planar Elongational Flows. J. Non-Newton. Fluid Mech..

[95] Flyvbjerg H., Petersen H.G. (1989). Error Estimates on Averages of Correlated Data. J. Chem. Phys..

[96] Colby R.H., Boris D.C., Krause W.E., Dou S. (2007). Shear thinning of unentangled flexible polymer liquids. Rheol. Acta.

[97] Stratton R.A. (1954). Non-Newtonian Flow in Polymer Systems with No Macromolecules Entanglement Coupling. Macromolecules.

[98] Yan Z.C., Costanzo S., Jeong Y., Chang T., Vlassopoulos D. (2016). Linear and Nonlinear Shear Rheology of a Marginally Entangled Ring Polymer. Macromolecules.

