# Supplementary material for: Scaling Relationships of the Structural and Rheological Behavior of Tadpole Polymer Chains in Dilute Solution Systems Using Brownian Dynamics Simulations
Source: Polymers (Basel). 2024 Oct 11;16(20):2871. doi: 10.3390/polym16202871 (PMC11510819; doi:10.3390/polym16202871)
Supplement: Supplementary file 1 [file polymers-16-02871-s001.zip › polymers-3232444-supplementary.pdf]

Supplementary Materials

Scaling Relationships of the Structural and Rheological Behavior of Tadpole Polymer Chains in Dilute Solution Systems Using Brownian Dynamics Simulations

Chaehyun Cho and Jun Mo Kim *

Department of Chemical Engineering, Kyonggi University, 154-42 Gwanggyosan-ro, Yeongtong-gu, Suwon 16227, Kyonggi-do, Republic of Korea; chaehyun@kyonggi.ac.kr
* Correspondence: junmokim@kgu.ac.kr; Tel.: +82-31-249-9045

Description of Movies.

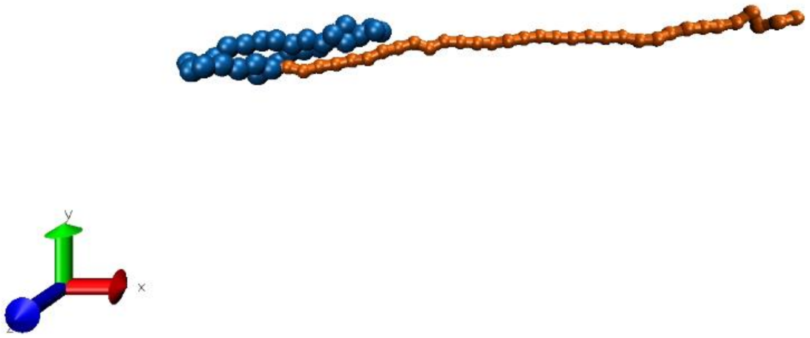

Movie S1. Chain rotation and tumbling behavior of T_R33L33 chain in the *xy*-plane at *Wi* = 1000 under shear flow.

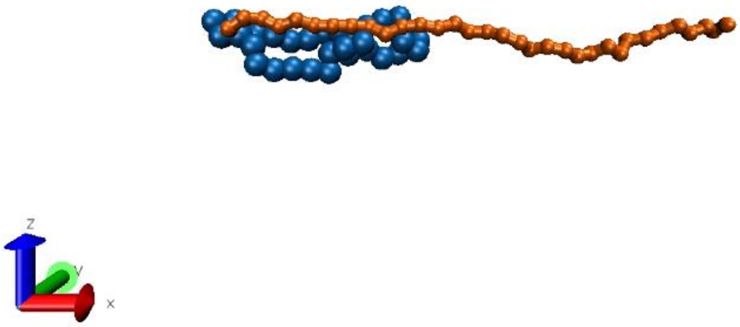

Movie S2. Chain rotation and tumbling behavior of T_R33L33 chain in the *xz*-plane at *Wi* = 1000 under shear flow.

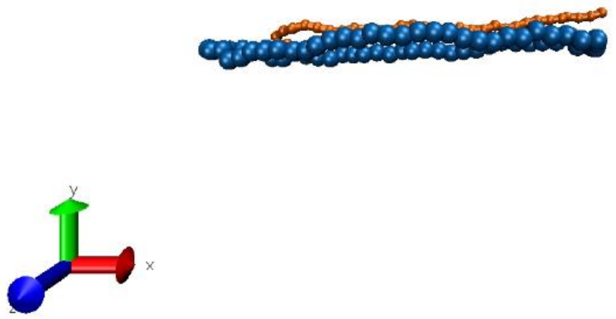

Movie S3. Chain rotation and tumbling behavior of T_R66L33 chain in the *xy*-plane at *Wi* = 1000 under shear flow.

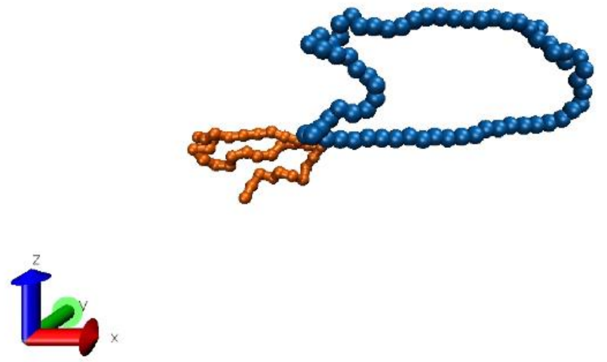

Movie S4. Chain rotation and tumbling behavior of T_R66L33 chain in the *xz*-plane at *Wi* = 1000 under shear flow.

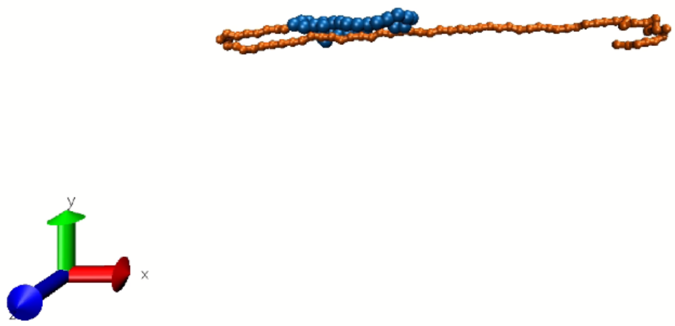

Movie S5. Chain rotation and tumbling behavior of T_R33L66 chain in the *xy*-plane at *Wi* = 1000 under shear flow.

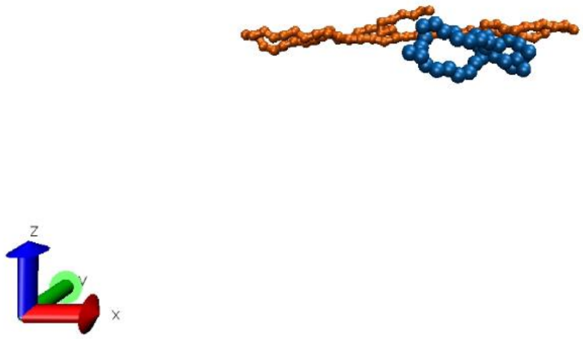

Movie S6. Chain rotation and tumbling behavior of T_R33L66 chain in the *xz*-plane at *Wi* = 1000 under shear flow.

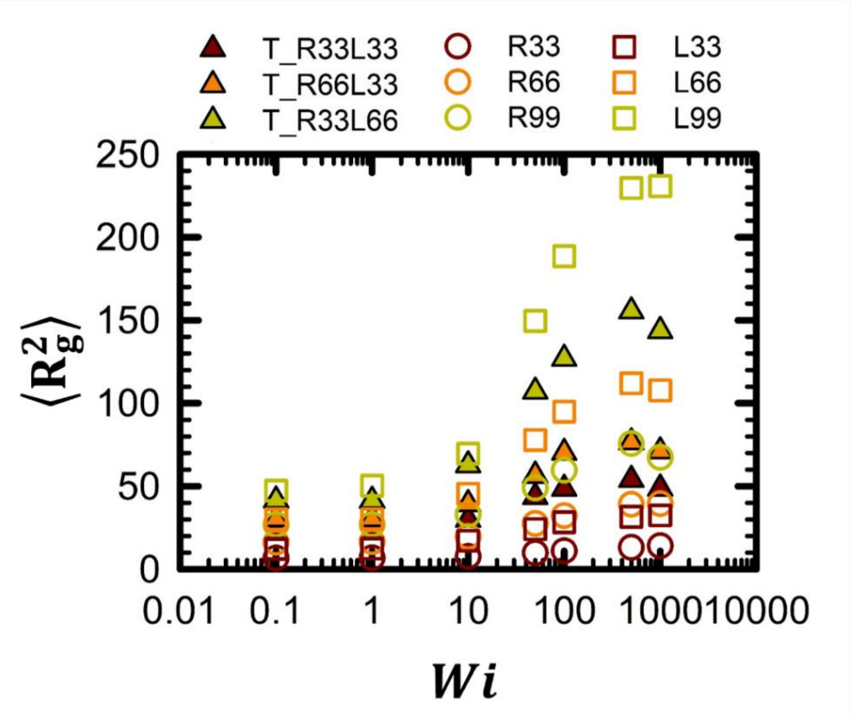

Figure S1. The mean square radius of gyration, $\langle R_g^2 \rangle$, for all tadpole polymer chains and corresponding linear and ring polymer chains as a function *Wi*.

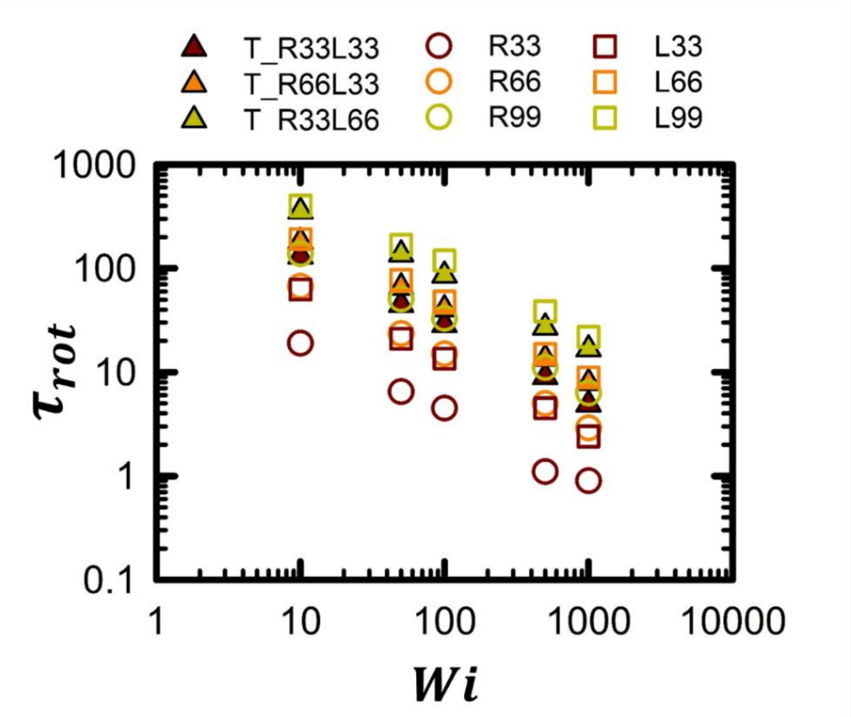

Figure S2. The rotational time, τ_{rot}, for all tadpole polymer chains and their linear and ring counterparts as a function of *Wi*. The rotational time is calculated by the time autocorrelation function of the unit chain end-to-end vector, $\boldsymbol{u}$.

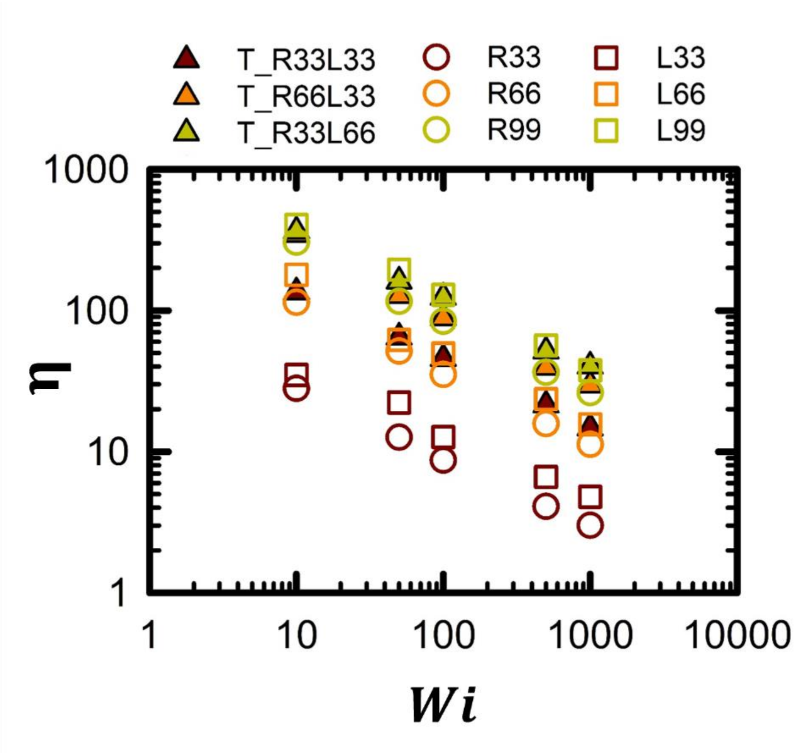

Figure S3. The shear viscosity, η, for all tadpole polymer chains and corresponding linear and ring polymer chains as a function of *Wi*.

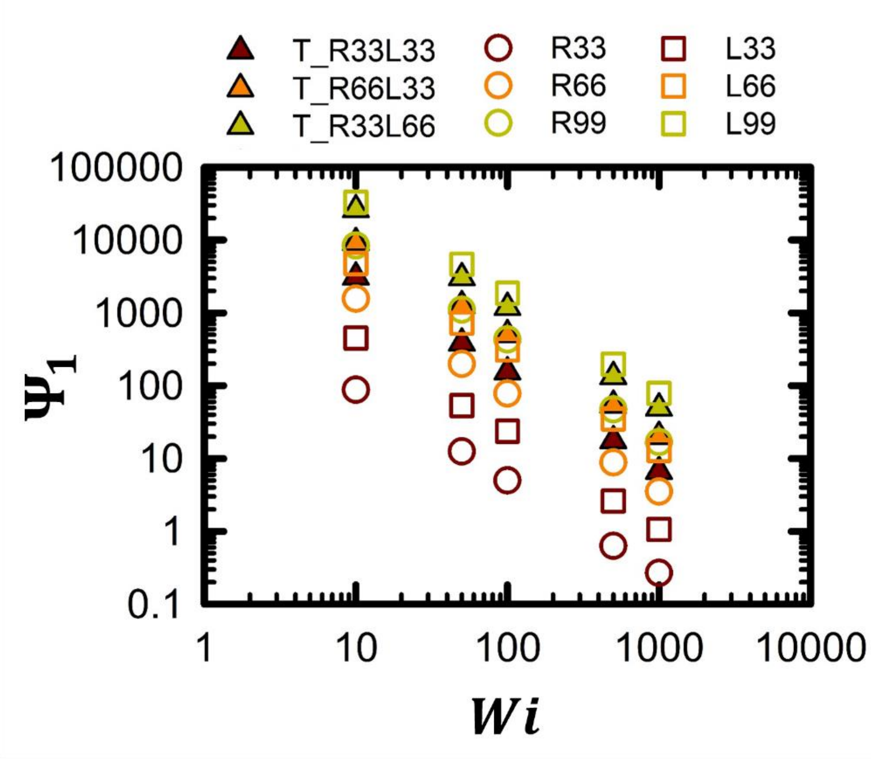

Figure S4. The first normal stress coefficient, Ψ_1, for all tadpole polymer chains and their linear and ring counterparts as a function of *Wi*.

Previous studies of tadpole-shaped polymer chains have been focused on the synthesis, purification, and characterization of tadpole-shaped polymer chains. In addition, the nonlinear flow behavior of tadpole-shaped polymer chains can be very challenging to observe in experiments because of the instability of the flow at high flow rates due to edge fracture, wall slip, and so on. To the best of our knowledge, there is insufficient research to elucidate the nonlinear viscoelastic response of tadpole polymer chains under flow conditions. Recent experimental study investigated the linear viscoelastic response of entangled tadpole polymer melt system [1]. However, due to inconsistencies in several test conditions (e.g., dilute solutions vs. melt system), a direct comparison with our simulation results appears difficult. Instead, we compare our simulated results with simulated and experimental results for unentangled linear and ring polymer chains in Figure S5 [2-4]. As can be seen in Figure S5, our simulation results for pure linear chains, pure ring chains, and tadpole polymer chains are in good agreement with the simulation and experimental results of the unentangled linear and ring polymer chain systems.

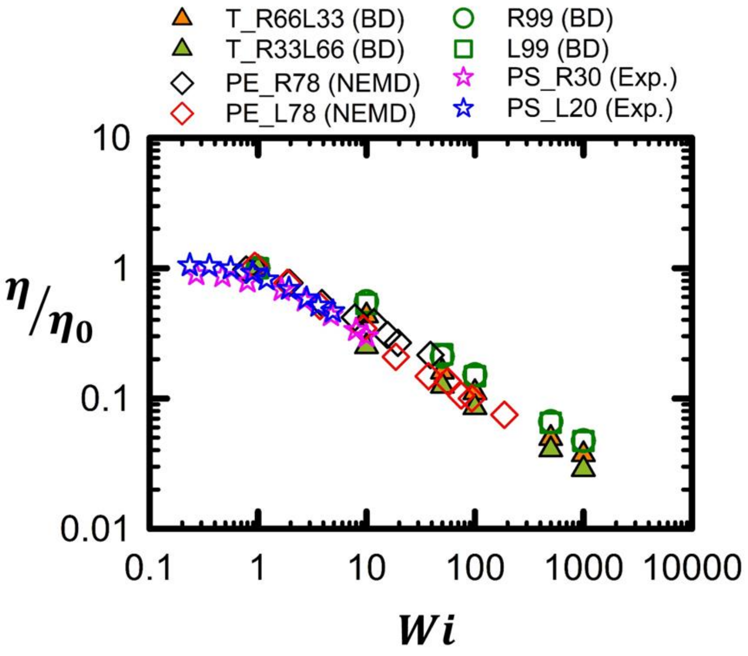

Figure S5. Comparison of the reduced viscosity of polymeric liquids obtained from simulation and experiment. Reproduced with permission from [2-4], published by Springer-Verlag, 2006, American Chemical Society, 1972, and The Society of Rheology, 2016, respectively.

We also compared our simulated data with the experimental data of the slightly entangled linear/ring blend melt systems [5]. In the experiments, the molecular weights of the pure linear chain, the pure ring chain, and the linear/ring blend systems were all the same, 84k. The 85:15 ratio of linear chain to ring chain is used in the linear/ring blend system. It should be noted that the linear/ring blend system under comparison here is not an unentangled chain system, but is a slightly entangled chain system with Z = 5 or 11 for linear polymer chains. As expected, the rheological properties of the slightly entangled linear/ring blend system were different from the rheological properties of the unentangled tadpole chain system. For example, in our study, the viscosity of unentangled tadpole polymer chain systems falls between the viscosity of the corresponding pure ring and pure linear chains at each shear rate. On the other hand, as shown in the Figure S6 a, the linear/ring blend melt system (black diamond) exhibits higher viscosity compared to corresponding pure linear (blue square) and pure ring (red circle) polymer chain systems. We can assume that the intermolecular interactions including complex topological constraints such as intermolecular ring-linear threading or intermolecular ring-ring threading are responsible for the increases in viscosity.

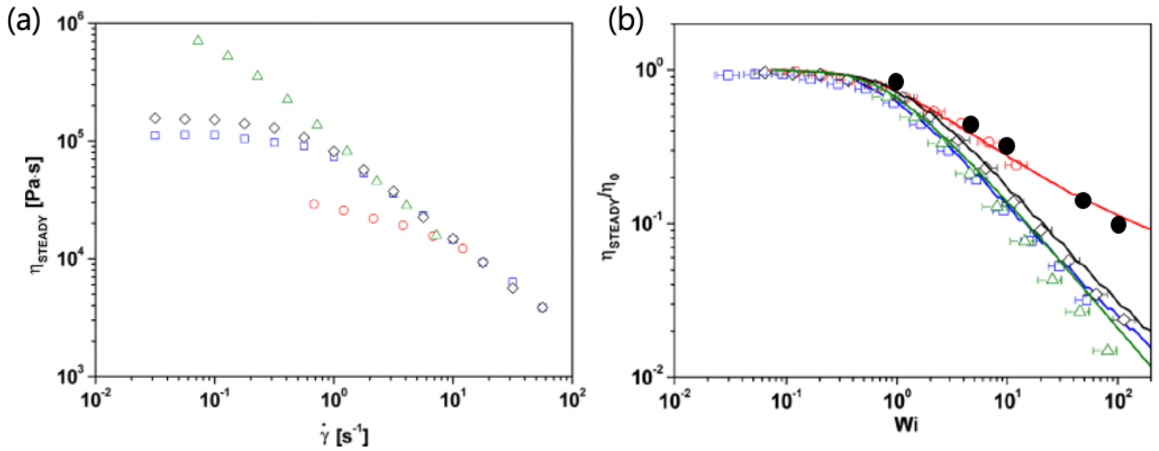

Figure S6. (a) Steady-state viscosity of PS rings, linear and blends vs shear rates (b) Steady viscosity normalized with the zero-shear viscosity as a function of *Wi*. Reproduced with permission from [5], published by American Chemical Society, 2016.

As can be seen in the Figure S6 b, the reduced viscosity of the unentangled tadpole polymer chains (black dot) is very similar to that of the pure ring polymer chain (red circle and red line) at each *Wi*. It is interesting to note that the power law exponent of *b*, which represents the

degree of shear thinning, is the same for both the unentangled tadpole chain system and pure ring system (b = -0.43 for the unentangled pure ring chain and -0.48 for the unentangled tadpole chain). A similar decreasing trend in reduced viscosity is observed for unentangled tadpole chain systems and unentangled pure ring chain systems without complex topological constraints. The linear/ring blend system with complex topological constraints decreases faster than the unentangled tadpole chain system and the pure ring system. We believe this implicitly demonstrates the impact of complex topological constraints on the rheological properties of linear/ring blend systems.

References

1. Doi, Y.; Takano, A.; Takahashi, Y.; Matsushita, Y. Melt Rheology of Tadpole-Shaped Polystyrenes. *Macromolecules* **2015**, *48*, 8667–8674.
2. Colby, R.H.; Boris, D.C.; Krause, W.E.; Dou, S. Shear thinning of unentangled flexible polymer liquids. *Rheol. Acta* **2007**, *46*, 569–575.
3. Stratton, R.A. Non-Newtonian Flow in Polymer Systems with No Macromolecules Entanglement Coupling. *Macromolecules* **1954**, *5*, 304-310.
4. Yoon, J.; Kim, J.; Baig, C. Nonequilibrium Molecular Dynamics Study of Ring Polymer Melts under Shear and Elongation Flows: A Comparison with Their Linear Analogs. *J. Rheol.* **2016**, *60*, 673–685.
5. Yan, Z.C.; Costanzo, S.; Jeong, Y.; Chang, T.; Vlassopoulos, D. Linear and Nonlinear Shear Rheology of a Marginally Entangled Ring Polymer. *Macromolecules* **2016**, *49*, 1444–1453.
